# Callus Induction from Diverse Explants and Genotypes Enables Robust Transformation of Perennial Ryegrass (*Lolium perenne* L.)

**DOI:** 10.3390/plants11152054

**Published:** 2022-08-05

**Authors:** Daniel Grogg, Marius Rohner, Steven Yates, Chloe Manzanares, Simon E. Bull, Sue Dalton, Maurice Bosch, Bruno Studer, Giovanni A. L. Broggini

**Affiliations:** 1 Molecular Plant Breeding, Institute of Agricultural Sciences, ETH Zurich, Universitaetstrasse 2, 8092 Zurich, Switzerland; 2 Institute of Biological, Environmental and Rural Sciences (IBERS), Aberystwyth University, Plas Gogerddan, Aberystwyth SY23 3EE, UK

**Keywords:** perennial ryegrass (*Lolium perenne* L.), *Agrobacterium*-mediated transformation, genome editing, functional genomics, doubled haploid (DH), tissue culture

## Abstract

Genetic transformation of perennial ryegrass (*Lolium perenne* L.) is critical for fundamental and translational research in this important grass species. It often relies on *Agrobacterium*-mediated transformation of callus tissue. However, callus induction is restricted to a few genotypes that respond well to tissue culture. Here, we report callus induction from different perennial ryegrass genotypes and explants, such as shoot tips, seeds, and anthers, which were transformed with several plasmids for functional genomics. β-glucuronidase (GUS) histochemical staining showed the *LmdsRNAbp* promoter sequence was active in stigmas, spikelets, anthers, and leaves. We also transformed calli with plasmids allowing gene silencing and gene knock-out using RNA interference and CRISPR/Cas9, respectively, for which genotypic and phenotypic investigations are ongoing. Using 19 different constructs, 262 transgenic events were regenerated. Moreover, the protocol regenerated a doubled haploid transgenic event from anther-derived calli. This work provides a proof-of-concept method for expanding the range of genotypes amenable to transformation, thus, serving research and breeding initiatives to improve this important grass crop for forage and recreation.

## 1. Introduction

Perennial ryegrass (*Lolium perenne* L.) is an important grass grown in temperate regions and is used for cattle grazing, feeding, and recreation (gardens, parks and golf courses, for example) [1]. Despite the agronomic and economic importance of perennial ryegrass, the genetic gain for fundamental traits, such as yield, lags behind that of other major crops like wheat, maize, and soybean [2,3]. The genetic gains in perennial ryegrass are low because of many factors. For example, the establishment of genomic resources for perennial ryegrass is still in its infancy, limiting the exploitation of genomics-based breeding approaches. Additionally, perennial ryegrass is an outbreeding species because of a genetically determined self-incompatibility system [4], which limits the use of more effective breeding strategies [2].

Nevertheless, genomic resources are increasingly becoming available in perennial ryegrass and closely related species [5,6,7]. Access to high-quality genome assemblies has many benefits; for instance, they are essential for genome-wide association studies, can simplify map-based cloning and also help discover candidate genes [8]. In short, they make research faster and easier. While genome assemblies are helpful, their gene models are mostly predictions, so in vivo gene function characterization and verification are needed. Various methods are available to study gene function in vivo, such as virus-induced gene silencing [9] and transient expression in protoplasts or leaves [10]. However, transient assays are limited to specific plant parts and development stages, making them unsuitable for genes that govern important agronomical traits.

In contrast, regenerating plants with stably expressed constructs offers broad utility and remains a valuable strategy for functional genomics. The stable integration of foreign DNA into the plant host genome allows studying gene function through several approaches, such as complementing with genes of interest (GOIs), downregulating expression levels of GOIs using RNA interference (RNAi), analyzing the expression patterns of GOIs using reporter systems or targeting GOIs using CRISPR/Cas systems, for instance [11]. Genetic transformation of crops (unlike the model species *Arabidopsis thaliana* (L.) Heyhn) requires an obligatory tissue culture step that represents a significant hurdle in many plant species, perennial ryegrass included [12]. Substantial efforts were invested in the genetic transformation of perennial ryegrass, as shown by the numerous publications reported by multiple independent research groups over the last three decades, which are summarized in Supplementary Table S1 [13,14,15,16,17,18,19,20,21,22,23,24,25,26,27,28,29,30,31,32,33,34,35,36,37,38,39,40,41,42,43,44,45,46,47]. Unfortunately, despite the availability of such a large number of reports and steady progress in this field, the overarching problem remains that *Lolium* transformation is a technically complicated and time-consuming procedure. Ultimately, genetic transformation relies upon callus induction, propagation, and regeneration of explants. However, the use of calli itself is a major limitation because of the genotype-dependent response to tissue culture reported in many monocotyledonous species [48].

Moreover, *Agrobacterium*-mediated transformation, the favorite gene transfer method in grasses [49], is also plagued by host-specific reactions that inhibit transformation efficiencies amongst genotypes [50]. Alternatively, biolistic transformation works with more genotypes, yet it is often impossible to regenerate plants after particle bombardment [12]. Besides the need for an amenable genotype, successful transformation depends on the origin, tissue, and organ material used to induce callus. For example, meristems excised from clones will generate genetically uniform calli, while calli derived from seeds or gametes will generate genetically diverse calli. Gamete-derived calli can regenerate completely homozygous individuals in a single generation, known as doubled haploids (DHs), which has advantages for self-incompatible species. Moreover, transforming gamete-derived calli, an unexplored alternative for perennial ryegrass, has the potential to regenerate individuals with homozygous transgenic events [51]. Therefore, using calli induced from multiple explants with distinct properties and from diverse germplasm is beneficial to a transformation platform as it offers increased flexibility and alleviates genotype-dependency. 

Here we report on the following: (1) callus induction from different genotypes and explant tissues, (2) an updated and complete *Agrobacterium*-mediated transformation protocol for perennial ryegrass, and (3) the successful transformation with *A. tumefaciens* carrying constructs allowing (β-glucuronidase) GUS reporter-aided analysis of promoter activities, knock-in, knock-down, and knock-out approaches for the functional study of genes of interest. For the optimization and the testing of the transformation protocol in perennial ryegrass, genes linked to self-incompatibility were targeted: *LpSDUF247-I*, *LpSDUF247-II*, and *LpdsRNAbp* representing genes within the *S*-locus [52,53], and *ZDUF247-I* and *ZDUF247-II* representing genes within the *Z*-locus [53,54]. Perennial ryegrass orthologs of a gene that orchestrates stomata development (*MUTE*) and one involved in the regulation of ethylene biosynthesis (*ETO1*) were also used as target genes [55,56]. Our callus induction and transformation protocol should facilitate the widespread use of transformation to advance functional genomics and breeding in perennial ryegrass, one of the most important forage and amenity grass species worldwide.

## 2. Results

### 2.1. Callus Induction and Agrobacterium-Mediated Transformation

**Figure 1:**
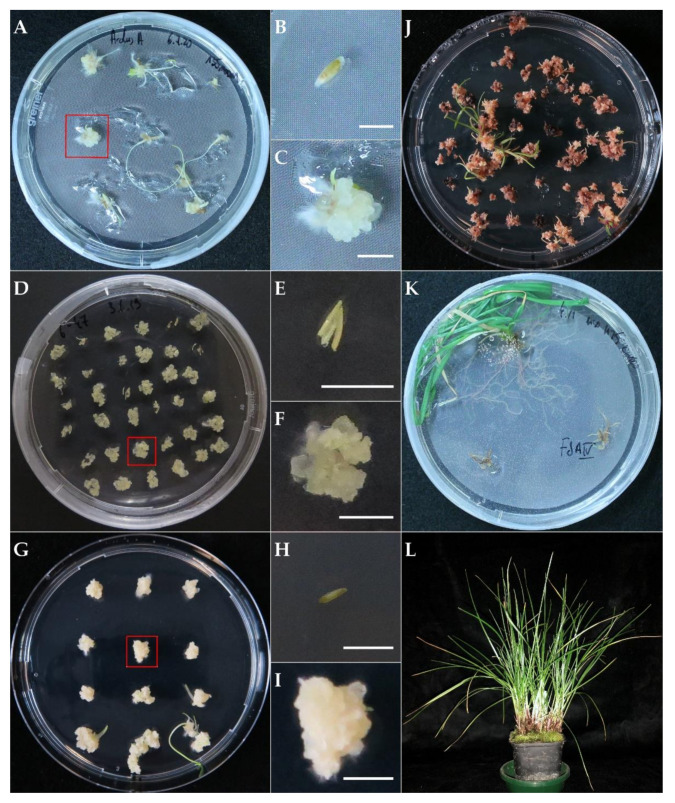
Representative plant tissue culturing steps for *Agrobacterium*-mediated transformation of perennial ryegrass (*Lolium perenne* L.). Petri dishes showing callus induction from seeds (**A**), and shoot tips (**G**) on media 135MODM, and anthers (**D**) on media R2M. Beside each panel, a close-up of a representative explant type prior to (**B**,**E**,**H**) and after callus induction (**C**,**F**,**I**) is shown. (**J**) Putative transgenic shoots regenerating from 135RMODM + H75 + C250 amongst untransformed calli affected by the selection; (**K**) putative transgenic plantlets rooted on MSO + H25 + C100 with two untransformed plantlets that perished through selection; (**L**) transgenic plant established in the soil. The scale bars in panels B, C, E, F, H, and I indicate 5 mm.

After four to six weeks post explant plating on callus induction media, calli were visible from the different types of explants: anthers, shoot tips and seeds (Figure 1). For the genotypes with characterized superior in vitro performances, CS128S23Z (hereafter referred to as S23 Z, meristem-derived) and 6–10 (anther-derived), calli appeared homogenous, meaning no further screening or selection was needed. In addition, the calli from genotypes S23 Z and 6–10 could be easily split and multiplied. In contrast, seed-derived calli from ‘Arolus’ displayed a more comprehensive range of phenotypes, and it was critical to isolate the best calli for downstream use at this stage. Therefore, only the fastest growing calli, derived from single seeds, displaying high spontaneous regeneration with low albinism frequency, were kept for subculture (i.e., callus lines). Out of 27 plated ‘Arolus’ seeds, three different seed-derived callus lines were generated and used for transformation: two for transformation with B330_MUTE2 and one with C801_GFP (Table 1). In all cases, generating enough material for the transformation required three to five rounds of subculture. During this process, unresponsive calli were weeded out. As the calli grew in size, the time between subculturing was shortened (at each stage) accordingly. Taken together, regardless of origin, the time from explant plating to transformable material lasted eleven to seventeen weeks. 

Transformation of calli derived from three explant types, with two different *Agrobacterium tumefaciens* strains carrying 19 different constructs, was successful (Table 1). Between one to nine plants were regenerated from the same callus, with plantlets regenerated from the same callus considered dependent (from the same event). Between 8.3% and 55.7% of the calli (median = 25.0%) of the clonal genotype S23 Z yielded an independent transgenic event following transformation (Table 1). 

In total, 320 putative transgenic plants, derived from 270 independent events, were regenerated and established in the soil (Table 1). A few additional albino plants were also regenerated from the transformation of seed-derived calli with construct B330_MUTE_2 but were discarded, as they cannot be established in the soil. Albino shoots were also observed with transformation of anther-derived calli with construct B330_ETO1_807 but failed to further develop into plantlets. During the selection process, short hairless roots developed from untransformed calli many times. In contrast, transformed calli formed long and hairy roots on rooting media (Figure 1K), making them easy to distinguish from untransformed calli.

### 2.2. T-DNA Characterization in Transgenic Plants

Out of the 320 putative transgenic plants screened, 304 plants regenerated from 262 independent events amplified a PCR amplicon confirming the integration of the corresponding selection marker *hptII* present on the T-DNA (Table 1). False–positive PCR amplification derived from residual *Agrobacterium* was excluded due to the lack of amplification of *Agrobacterium virD2* virulence gene when tested in a subset of 36 transgenic plants (data not shown) [57]. This subset consisted of transgenic plants regenerated from seed-derived calli transformed with the two constructs B330_MUTE_2 and C801_GFP and shoot tip-derived calli transformed with C801_GFP. Additionally, the entire T-DNA was Sanger-sequenced in a subset of ten plants transformed with constructs G150-G159 (i.e., one plant for each construct), confirming the correct integration of the full T-DNA (data not shown). A total of 16 escapes (i.e., plants that were able to survive the antibiotic selection but were negative to PCR amplification of the plant selection marker) regenerating from eight independent transformation events were discovered. These escapes were regenerated from the transformation of anther- and seed-derived calli with constructs C801_GFP (two plants from one event), B330_ETO1_807 (one plant from one event), and B330_MUTE_2 (13 plants from six events), respectively. No escapes regenerated from the transformation of shoot tip-derived calli with the other constructs (Table 1).

### 2.3. Genes Activity in Transgenic Lines

**Figure 2:**
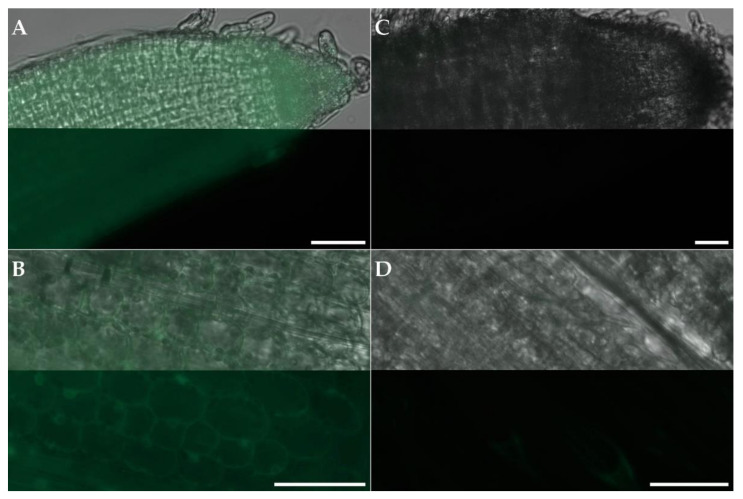
Fluorescence microscopy pictures of the root tip (**A**) and leaf segment (**B**) of a transgenic perennial ryegrass (*Lolium perenne* L.) plant regenerated from the transformation of ‘Arolus’ seed-derived calli with GFP expression driven by the maize ubiquitin promoter (C801_GFP); root tip (**C**) and leaf segment (**D**) of an untransformed plant displaying no GFP expression. In all panels, the top part shows an overlay of GFP and brightfield channels and the bottom part GFP channel only. The scale bars indicate 50 µm.

Examination of GFP activity by fluorescence microscopy showed a constitutive expression of GFP driven by the maize ubiquitin promoter (p*Zm*Ubi-1) in seven transgenic plants transformed with C801_GFP (Table 2). The difference was evident between transformed lines and negative controls (untransformed), as shown in Figure 2.

**Figure 3:**
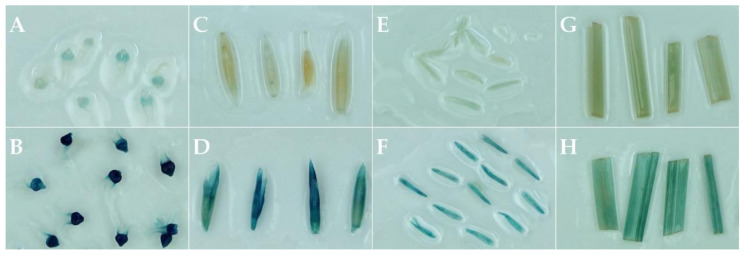
GUS staining of a perennial ryegrass (*Lolium perenne* L.) transgenic plant regenerated from the transformation of S23 Z shoot tip-derived calli transformed with the promoter sequence of the *LmdsRNAbp* fused to GUS (G160). Different tested tissues include stigmas (**A**,**B**); spikelets (**C**,**D**); anthers (**E**,**F**); and leaves (**G**,**H**). The top panels (**A**,**C**,**E**, and **G**) show the negative control (untransformed), and the bottom panels (**B**,**D**,**F**, and **H**) a transgenic plant transformed with G160.

GUS histochemical staining of stigmas, spikelets, anthers, and leaves of plants transformed with the construct G160 indicated that the 1500 bp promoter sequence of *LmdsRNAbp* induced *GUS* expression in all these organs, with the strongest GUS activity in stigmas and spikelets (Figure 3, Table 2). Weak endogenous GUS-like activity was detected in stigmas and anthers of the untransformed controls, but GUS-like activity was undetected in spikelets and leaves (Figure 3). Plants transformed with construct G166 (GUS driven by a CaMV 35S promoter) showed GUS activity in leaf samples for two independent lines tested, with no activity observed in untransformed control (data not shown). Failure to vernalize G166 lines meant only leaves were tested for GUS histochemical staining. For the plants transformed with the 1500 bp promoter sequence of *LmSDUF247-I* (G161), *LmSDFU247-II* (G162), *LmZDUF247-I* (G163), and *LmZDUF247-II* (G164) fused to GUS, a total of 20 independent lines (G161: 5, G162: 5, G163: 6, G164: 4) were successfully vernalized, allowing assessment of the promoter activity through GUS histochemical staining in different tissue types (stigmas, spikelets, anthers, and leaves). No apparent GUS activity could be observed in any tissue type for transgenic plants harboring G161–G164 (data not shown).

Preliminary PCR-based genotyping of the loci targeted by CRISPR/Cas9 constructs revealed low editing efficiency shortly after the establishment of the plants in the soil. The presence of a heterozygous insertion of one adenine was observed and confirmed by Sanger-sequencing in a single transgenic line out of the 70 B330_MUTE_2, while no edits were observed in B330_ETO1_807 transgenic lines (Supplementary File S1). RT-qPCR analysis confirmed *Cas9* transcription in the transgenic lines using the Pfaffl method [58] with *EF1α* [59] as a reference gene.

## 3. Discussion

Here we report a complete protocol for callus induction from different perennial ryegrass genotypes and explants and their genetic transformation. This protocol can be applied to further broaden the range of genotypes to be transformed for functional genomics and crop improvement. The reported transformation protocol is based on the protocols developed by Dalton [42] and Begheyn et al. [60] and is robust in yielding transgenic perennial ryegrass plants. The reported work was conducted at two different locations: experiments with S23 Z in Aberystwyth, Wales (UK), and those with ‘Arolus’ and 6–10 in Zurich, Switzerland. 

The three different explants used in this study each had their specific trade-offs. The large number of transgenic lines obtained from shoot tips of S23 Z confirmed its outstanding suitability for callus induction and transformation. However, working with shoot tips was laborious: maintenance of in vitro clones was required to make shoot tips available throughout the year and delicate meristem excision was required for callus induction. Additionally, the cultivar S23, of which S23 Z is a genotype, is no longer considered an elite cultivar. Using seeds was the easiest approach to induce calli as prior treatment was unnecessary. Seeds were easy to store and were available in large numbers. Yet, each seed was genetically different, thus leading to varying in vitro responses within the same cultivar. Ergo, the seed method could effortlessly be upscaled to screen the seeds of any cultivar, whereby only responsive genotypes are kept. In contrast, callus induction from anthers was the most difficult approach: anther availability was limited to a few weeks per year and needed prior vernalization of the plants to induce flowering. Once plants flowered, harvesting anthers at the right developmental stage was delicate and time-consuming. Furthermore, for many genotypes setting up cultures for anthers is challenging. The protocol is likely to fail unless the genotypes being used are known to respond well to anther culture conditions (e.g., the androgenic line 6–10 and others reported by Begheyn et al. [60]). However, anther cultures can regenerate DH lines, which are advantageous for self-incompatible species. Taken together, we found S23 Z shoot tips were the most reliable for callus induction and subsequent transformation. However, if the research goal is to explore agronomical properties in elite cultivars, we recommend attempting callus induction from seeds because of the above-mentioned reasons.

Most publications reporting perennial ryegrass transformation experiments do not report quantification of callus induction and regeneration efficiency. If reported, this was assessed using different methods. For example, Dalton [42] used the callus weight, plants per gram of callus, and plants per explant to quantify callus induction and plant regeneration. Zhang et al. [43], however, mentioned embryogenic callus lines with “outstanding regeneration ability” without giving further detail on how the regeneration ability was assessed.

Comparing the efficiency of the transformation protocol presented here with those reported by others (Supplementary Table S1, [13,14,15,16,17,18,19,20,21,22,23,24,25,26,27,28,29,30,31,32,33,34,35,36,37,38,39,40,41,42,43,44,45,46,47]) is challenging. Transformation efficiencies have been assessed using different methods. Patel et al. [34] used percentage of callus expressing GFP and not regenerated plants. Altpeter et al. [18] used the number of calli, and independent transgenic plants regenerated thereof. Bajaj et al. [26] did the same without mentioning whether the plants were regenerated from dependent or independent events. Zhang et al. [35] reported the number of callus lines that generated GFP plants versus the number of callus lines. Cao et al. [27] assessed transformation efficiency by comparing the number of plants regenerated on selective media versus the number of plants regenerated without selection. In summary, a lack of consensus on how transformation and regeneration efficiencies should be measured effectively hampers direct comparison between different studies, and this is a situation we also faced with our manuscript. However, the protocol implemented in this study incorporated the following three factors previously reported to improve transformation in monocotyledonous species, ryegrass included [34,43]: (1) including myo-inositol in the media only after *Agrobacterium* infection, (2) heat-shock applied prior to the infection and, (3) co-cultivation on high maltose concentration. 

Various hygromycin concentrations and selection regimes were applied during selection. With S23 Z calli, hygromycin concentrations between 75–80 mg·L^−1^ were used for callus selection, regeneration and rooting. With ‘Arolus’ and 6–10 calli, hygromycin concentration was gradually increased from 25 to 75 mg·L^−1^ during callus selection, reduced to 50 mg·L^−1^ for regeneration and to 25 mg·L^−1^ for rooting. The higher selection pressure applied to S23 Z calli hindered the regeneration of escape plants, which were observed when applying a lower selection pressure to ‘Arolus’ and 6–10 calli. Therefore, to minimize escapes, the highest selection pressure showing minimal toxicity in transgenic plants should be applied during the selection steps, including rooting. 

A common issue in the tissue culture of monocotyledonous species is albinism, which is under genetic and environmental influence [61]. Due to the absence of photosynthetic activity, albino plants cannot survive outside of tissue culture. Multiple albino plantlets were regenerated after transformation of ‘Arolus’ seed-derived and 6–10 anther-derived calli but not with S23 Z shoot tip-derived calli. Albino regenerants in the former two genotypes were also observed prior to transformation (data not shown). Carefully selecting calli with a minimal albinism rate during the callus preparation steps should reduce the occurrence of albinism in transgenic regenerants. 

Transgenic perennial ryegrass lines expressing reporter proteins were established. The *Zm*Ubi-1 promoter was found to drive expression of GFP in roots and leaves, while the *LmdsRNAbp* promoter drove expression of GUS in leaves, anthers, stigmas, and spikelets. While Manzanares et al. [52] showed the activity of the *LpSDUF247-I* in pollen, our experiments did not confirm transcriptional activity of the 1500 bp promoter sequence upstream of *LmSDUF247-I*. The reasons could be an insufficient expression of GUS by the promoter (for GUS detection), silencing of the constructs, or mismatch between tissue type and time point during flowering.

To our knowledge, this is the first reported transformation of anther-derived calli and regeneration of transgenic DH plants in perennial ryegrass. Preliminary flow cytometry data confirmed that transgenic plants were diploid while microsatellite marker analysis revealed homozygous allelic composition (data not shown), confirming the DH status of the transgenic plants. However, further tests are required to determine the zygosity of the T-DNA integration; for instance, by investigating transgene segregation in the progeny of these plants. Homozygous T-DNA integration has been achieved in wheat and barley by transforming microspore cultures prior to callus induction [51,62] or upon haploid callus formation [63]. In our case, we transformed anther-derived calli and whether they had already undergone chromosome doubling or still contained haploid cells was unknown. In self-incompatible species like perennial ryegrass, this would enable homozygous T-DNA integration within a single generation, thus, circumventing tedious successive rounds of selfing otherwise required and would represent a major advance.

The molecular and phenotypic characterization of the RNAi lines (constructs G150 to G159) was beyond the scope of this manuscript. The gene targets of the RNAi lines are involved in the reproductive system of perennial ryegrass. They are exclusively expressed during flowering in the pollen tissue. The phenotypic and genotypic characterization of the RNAi lines requires vernalization and repeated experiments over multiple flowering seasons. This multiple-year research project could not be adequately reported within this manuscript, which describes the palette of options available for successful callus induction and its genetic transformation.

Characterization of the CRISPR lines indicated a low editing efficiency, and the confirmed expression of *Cas9* in transgenic plants excluded post-transcriptional gene silencing as a possible cause of the low editing efficiency. Editing events may accumulate and get fixed in the plant material over the time. Thus, further efforts are required to screen all the transgenic lines produced in this study at a later stage.

This protocol was shown to be successful in transforming calli from different explants and genotypes at two different locations, emphasizing its robustness. Such a protocol would facilitate functional genomics studies in perennial ryegrass. This knowledge would be useful for improvement of this major grass crop used for forage and recreation.

## 4. Materials and Methods

### 4.1. Plant Growth Conditions

#### 4.1.1. Induction of Flowering

To establish flowering plants for callus induction from 6–10 anthers and to observe GUS activity in flowers of transgenic S23 Z plants harboring a promoter GUS construct (G160-G164 and G166, Table 2), plants were vernalized at 4 °C under short-day conditions (8 h light: 16 h dark) for 14 to 20 weeks. After vernalization, plants were transferred into a climate chamber under long-day conditions (16 h light: 8 h dark) with temperatures ranging from 20 to 24 °C and 60% relative humidity. Plants started flowering after four to ten weeks of long-day conditions, and inflorescences were harvested and prepared for anther culture, as described in Begheyn et al. [60], or GUS histochemical staining.

#### 4.1.2. Establishment in the Soil

Single tillered plantlets that rooted successfully in vitro were transferred to individual 2 L pots filled with a soil perlite mixture. Once established in soil, the plants were grown in growth chambers under long-day conditions (16 h light: 8 h dark) with temperatures ranging from 18 to 22 °C, and 60% relative humidity. 

### 4.2. Callus Induction from Various Explants and Genotypes

Calli were induced from anthers of the androgenic perennial ryegrass genotype 6–10 (DLF A/S, Store Heddinge, Denmark) and shoot tips of the perennial ryegrass genotype S23 Z [64], according to Begheyn et al. [60] and Dalton [42], respectively. Furthermore, the perennial ryegrass elite cultivar ‘Arolus’ (Agroscope, Reckenholz, Switzerland) was used to induce calli from seeds following the protocol described in the supplementary data (Supplementary File S2). After four to six weeks, the developed calli were subcultured at low density (a maximum of twelve calli per 100 × 15 mm petri dish) onto fresh 135MODM medium (Supplementary File S2) to promote fast growth and generate sufficient callus for the transformations. The time interval between each subculture was gradually reduced (e.g., subculture onto fresh 135MODM after four, three, and two weeks). Following these principles, different subculture regimes were applied, based on the growth pattern of each culture. Irrespective of their origin, all calli were subcultured for the last time one week prior to the transformation date to ensure active cell division.

### 4.3. Agrobacterium-Mediated Transformation

*Agrobacterium*-mediated transformation was adapted from Dalton [42], as described in detail in the supplementary methods (Supplementary File S2). ‘Arolus’ seed-derived calli were transformed with *A. tumefaciens* strain GV3101:pMP90RK carrying the construct C801_GFP and B330_MUTE_2 (Table 2). S23 Z shoot tip-derived calli were transformed with strain *A. tumefaciens* LBA4404 and GV3101:pMP90RK carrying the hairpin-based RNAi, promoter GUS, and GFP constructs (Table 2). The *A. tumefaciens* strain GV3101:pMP90RK carrying construct B330_ETO1_807 was used to transform 6-10 anther-derived calli (Table 2).

### 4.4. Plasmid Construction and Transformation into A. tumefaciens

A total of 19 constructs were prepared using different cloning methods and backbones for *Agrobacterium*-mediated transformation, a summary of which is provided in Table 2 and described below. The prepared constructs were transformed into different *A. tumefaciens* strains (Table 2) by electroporation [65] and plated onto lysogeny broth agar plates supplemented with adequate antibiotics, before single colonies were picked and glycerol stocks prepared.

### 4.5. Constructs Preparation

#### 4.5.1. Creation of the Hairpin-Based RNAi Constructs

**Figure 4:**
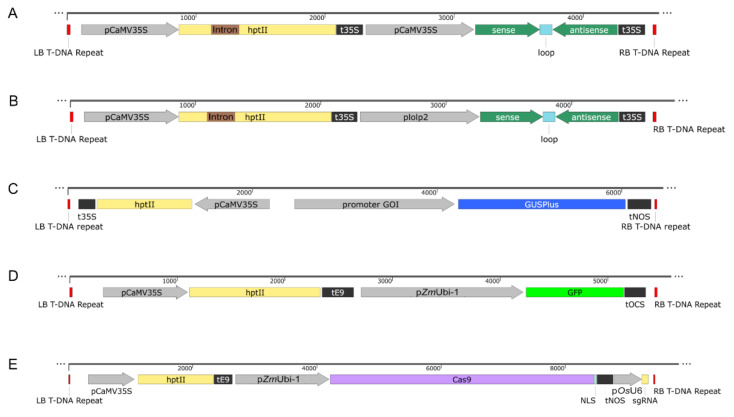
Structural organization of the T-DNA of the binary vectors for *Agrobacterium*-mediated plant transformation used in this study. All constructs (**A**–**E**) contained a hygromycin B resistance (*hptII*) for plant selection. (**A**,**B**) Composition of the hairpin-based RNAi (G150-G159) constructs where the hairpin targeting the gene of interest (GOI) was either driven by a CaMV 35S promoter (A: G150, G152, G154, G156, and G158) or by the lolp2 promoter (B: G151, G153, G155, G157, and G159). The ten hairpin-based RNAi constructs harbored a CaMV 35S terminator signaling the termination of the transcription. (**C**) Composition of the promoter-GUS expression constructs harboring the promoter region of the GOI fused to GUS (G160-G164) with a NOS terminator. (**D**) Composition of the GFP construct with a maize ubiquitin promoter (pZmUbi-1) driving the expression of the GFP with an tOCS terminator. (**E**) Composition of the CRISPR constructs (B330_MUTE_2 and B330_ETO1_807) with pZmUbi-1 driving the expression of the *Cas9* fused to a nuclear localization signal (NLS). The transcription was terminated through a NOS terminator. The single guide RNA (sgRNA) that defined the genetic target for modification, either *LpMUTE* or *LpETO1*, was driven by a rice U6 promoter (pOsU6). The left (LB) and right border (RB) of the T-DNA region is displayed (LB T-DNA repeat and RB T-DNA repeat).

In the diploid genome assembly of Italian ryegrass (*Lolium multiflorum* Lam.) ‘Rabiosa’ from Copetti et al. [6], the gene sequences for *dsRNAbp, SDUF247-I, SDUF247-II*, *ZDUF247-I,* and *ZDUF247-II* were extracted (Table 3) [52,53,54]. If no annotation was present for the GOI, the gene structure was added using Augustus (Organism: *Oryza brachyantha*) gene prediction tool [66] or manually using a BLAST-based approach. Through a PCR assay designed for targeted resequencing, the orthologous allele sequences were acquired in S23 Z. For each of the five GOIs, a hairpin was designed with a size of 500 bp according to the following criteria: specificity for the GOI (no off-targets) and silencing both alleles of a single target gene. The possible presence of off-targets was evaluated in the genome assembly of perennial ryegrass P226/135/16 [67] and the genome assembly of Italian ryegrass ‘Rabiosa’ [6]. A syntenic plant intron sequence (GenBank: M27939.1, REGION: 71..165) was used as a loop sequence. The sense sequence of the hairpin combined with the loop sequence and an Xhol restriction site at the 3′-end were combined using Benchling^TM^ and was named sense_cloning for simplicity. Similarly, the antisense sequence was combined with an Xhol restriction site at the 5′-end and was named antisense_cloning for simplicity. The sense_cloning and antisense_cloning sequences were ordered individually as gene strings using the GeneArt™ Services (Thermo Fisher Scientific, Waltham, MA, USA). The sequences were blunt-end cloned into pjet1.2 (Thermo Fisher Scientific, Waltham, MA, USA) and transformed into chemically competent *E. coli* TOP10 cells. The purified plasmids were digested with the restriction enzymes XhoI and BpiI (Thermo Fisher Scientific, Waltham, MA, USA). The released target fragments were separated with a 1 × TAE 1% agarose gel electrophoresis, and the DNA bands matching the expected size were cut and column cleaned with the Wizard^®^ SV Gel and PCR Clean-Up System, following the manufacturer’s protocol (Promega, Madison, WI, USA). The multigene binary cassettes were assembled using the Golden Gate cloning system [68,69]. The two cleaned-up sequences (sense_cloning and antisense_cloning) that constituted the hairpin were ligated into a BpiI digested Level 0 module (pICH41308) and were confirmed by Sanger-sequencing (Microsynth AG, Balgach, Switzerland). The primers used for sequencing the level 0 modules (pICH41308) harboring the hairpin against the GOIs (*LpdsRNAbp*, *LpSDUF247-I*, *LpSDUF247-II*, *LpZDUF247-I*, *LpZDUF247-II*) were 153_pL0-seq-F and 154_pL0-seq-R (Supplementary Table S2). BpiI restriction sites were added to the promoter region of the *L. perenne* pollen-specific *Lol p 2* gene (GenBank: AY533648.1, [22]) sequence (plolp2) and was subsequently cloned into a level 0 acceptor module (pICH41295) using Golden Gate cloning [68,69]. Positive colonies harboring the lolp2 promoter in the level 0 acceptor module (pICH41295) were further confirmed by Sanger-sequencing with the primer 153_pL0-seq-F and 154_pL0-seq-R (Supplementary Table S2) (Microsynth AG, Balgach, Switzerland). Following the Golden Gate cloning procedure, the hairpin expressing cassette was assembled in a level 1 acceptor (pICH47742, position 2, forward orientation). Two different cassettes were assembled for each GOI (*LpdsRNAbp*, *LpSDUF247-I*, *LpSDUF247-II*, *LpZDUF247-I*, *LpZDUF247-II*); either the CaMV 35S promoter double (pICH51288) or the lolop2 promoter was driving the hairpin expression. The CaMV 35S terminator (pICH41414) was used for all constructs as a terminator. The cassette required for plant selection expressing the hygromycin B resistance (*hptII*) gene with a CaMV 35S promoter and terminator sequences (L1-F1-p35S-*hptII*-t35) was described by Bull et al. [70]. Each of the ten different Level 1 acceptors (pICH47742) harboring the cassette for the expression of the hairpins at position two forward in combination with the L1-F1-p35S-*hptII*-t35 at position one forward harboring plant selection cassette, End-link 1 (pICH49255), and End-link 2 (pICH4174) were assembled into the Level 2 acceptor (pAGM4673) following the Golden Gate cloning procedure. The T-DNA of the level 2 acceptor (pAGM4673) was Sanger-sequenced (Microsynth AG, Balgach, Switzerland) with the sequencing primers 173_RB_L2_seq, 174_LB_L2_seq, 175_hptII-seq-F, 176_hptII-seq-R, 177_posthptII_F 178_posthptII_R, 179_sense35S_seq (specific for CaMV 35S promoter-driven hairpin constructs) and 180_senselolp2_seq (specific for lolp2 promoter-driven hairpin constructs) (Supplementary Table S2). Table 2 summarizes the ten final (G150-G159) binary vectors for *Agrobacterium* T-DNA delivery to plant cells. Two representative T-DNAs of the hairpin-based RNAi constructs are displayed in Figure 4A,B. 

#### 4.5.2. Creation of Promoter GUS Constructs

The promoter region of the *dsRNAbp*, *SDUF247-I*, *SDUF247-II*, *LpZDUF247-I*, and *ZDUF247-II* was identified within a diploid genome assembly of Italian ryegrass ‘Rabiosa’ [6] (Table 3). The promoter region was defined as 1500 bp upstream of the start codon. Primers were designed to amplify the identified promoter region of interest (*dsRNAbp*, *SDUF247-I*, *SDUF247-II*, *ZDUF247-I*, and *ZDUF247-II*, Table 3) and to simultaneously add a HindIII restriction site at the 5’-end and a BglII restriction site at the 3′-end (Supplementary Table S2). The primers used for the amplification of the five promoter regions of interest were as follows: *LmdsRNAbp* (163_dsRNAbp-F and 164_dsRNAbp-R), *LmSDUF247-I* (165_SDUF247-I-F and 166_SDUF247-I-R 2), *LmSDUF247-II* (167_SDUF247-II-F and 168_SDUF247-II-R), *LmZDUF247-I* (169_ZDUF247-I and 170_ZDUF247-I-R), and *LmZDUF247-II* (171_ZDUF247-II and 172_ZDUF247-II-R) (Supplementary Table S2). Following the manufacturer’s protocol, the PCR was performed with the Q5^®^ High-Fidelity DNA Polymerase (New England Biolabs, Frankfurt, Germany). Cycling conditions were 30 s at 98 °C, followed by 35 cycles of 10 s at 98 °C, 30 s at 61 °C and 50 s at 72 °C, and a final elongation step at 72 °C for 1 min. The PCR amplicons were separated with a 1 × TAE 1% agarose gel electrophoresis, and the DNA bands matching the expected size were cut and column cleaned with the Wizard^®^ SV Gel and PCR Clean-Up System following the manufacturer’s protocol (Promega, Madison, WI, USA). Subsequently, the cleaned-up amplicon sequences were blunt-end cloned into pjet1.2 (Thermo Fisher Scientific, Waltham, MA, USA) and transformed into chemically competent *E. coli* TOP10 cells. The five pjet1.2 vectors harboring the promoter region of the GOI and the pCAMBIA1305.1 (CAMBIA) were digested with HindIII and BglII (Thermo Fisher Scientific, Waltham, MA, USA). The amplicon and vector fragments were separated with a 1 × TAE 1% agarose gel electrophoresis, and the DNA bands matching the expected size were cut and column cleaned with the Wizard^®^ SV Gel and PCR Clean-Up System following the manufacturer’s protocol (Promega). The linearized vector pCAMBIA1305.1 was ligated with the promoter sequence of the GOI with a T4 DNA ligase (Thermo Fisher Scientific, Waltham, MA, USA). The ligation mix was used to transform chemically competent *E. coli* TOP10 cells. Positive plasmids were Sanger-sequenced (Microsynth AG, Balgach, Switzerland) using the primers 184_pC1305.1_MSC-F and 185_pC1305.1_MSC-R (Supplementary Table S2). Following standard cloning procedure, five multigene cassettes consisting of the promoter of interest fused to GUS, and a hygromycin B resistance (*hptII*) for plant selection were assembled in pCAMBIA1305.1. The GUS constructs (G160-G164) and the unaltered pCAMBIA1305.1 (G166, CaMV35S promoter driving the GUS expression) are summarized in Table 2. The T-DNA of the promoter GUS constructs were Sanger-sequenced (Microsynth AG, Balgach, Switzerland), and a representative construct is shown in Figure 4C.

#### 4.5.3. Acquisition of the GFP Construct 

The GFP construct (C801_GFP) was ordered from DNA cloning service (Hamburg, Germany) and is summarized in Table 2. The T-DNA harbors a hygromycin B resistance (*hptII*) for plant selection and a GFP cassette. The GFP cassette consisted of a *Zm*Ubi-1 promoter driving the expression of GPF and an octopine synthase gene terminator (tOCS), as displayed in Figure 4D.

#### 4.5.4. Creation of CRISPR Constructs

The orthologous gene sequence of *MUTE* [55] from *Brachypodium distachyon* was identified within the diploid genome assembly of Italian ryegrass ‘Rabiosa’ [6] (Table 3). The perennial ryegrass *ETHYLENE OVERPRODUCER1* (*LpETO1*) gene sequence was previously identified by Manzanares et al. [71], and the orthologous gene sequences were extracted in Italian ryegrass ‘Rabiosa’ (Table 3). *LpMUTE* and *LpETO1* were amplified with the Q5^®^ High-Fidelity DNA Polymerase (New England Biolabs, Frankfurt, Germany), following the manufacturer’s protocol with MUTE_F1 and MUTE_R3 and ETO1_F and ETO1_R primers, respectively, in *L. perenne* ‘Arolus’ and 6-10 (Supplementary Table S2). For *LpMUTE*, cycling conditions were 30 s at 98 °C, followed by 35 cycles of 10 s at 98 °C, 30 s at 66 °C and 30 s at 72°C, and a final elongation step at 72 °C for 2 min. For *LpETO1*, cycling conditions were 30 s at 98 °C, followed by 35 cycles of 10 s at 98 °C, 30 s at 63 °C and 40 s at 72 °C, and a final elongation step at 72 °C for 2 min. The obtained PCR products were then Sanger-sequenced with MUTE_R3 (*LpMUTE*) or ETO1_F (*LpETO1*) primers (Supplementary Table S2). Sequences were aligned using MAFFT algorithm [72], and target-specific spacers were designed on regions of the first exons conserved amongst both genotypes. Twenty nucleotide long spacers were designed in Benchling^TM^ using the *L. perenne* genome [67], and for each target, one spacer with an efficiency score ≥ 60 [73] was selected. A supplementary G was included at the 5′ end of both spacers to facilitate their transcription by the OsU6 promoter. Two plasmids carrying target-specific spacer for Cas9 mutagenesis in *LpMUTE* and *LpETO1* were then developed. For this, two synthesized 600 bp long DNA fragments (Life Technologies Europe B.V., Zug, Switzerland) containing the OsU6 promoter and sgRNAs (MUTE_2 and ETO1_807 specific spacers previously designed and gRNA scaffold) were amplified using Q5^®^ high-fidelity DNA polymerase (New England Biolabs, Ipswich, MA, USA) with ETO1_stringF and ETO1_stringR primers containing EcoRI and SalI restriction sites, respectively (Supplementary Table S2). Cycling conditions were 30 s at 98 °C, followed by 30 cycles of 10 s at 98 °C, 30 s at 65 °C and 30 s at 72 °C, and a final elongation step at 72 °C for 2 min. B330p6i2xoR-UcasW-pOsU6 (DNA cloning service, Hamburg, Germany), hereinafter referred to as B330, and the purified PCR products were doubled digested with EcoRI HF and SalI HF (New England Biolabs, Ipswich, MA, USA). They were then ligated using T4 DNA Ligase (New England Biolabs, Ipswich, MA, USA), following the manufacturer’s instructions. The obtained constructs were Sanger-sequenced with RB_rev primer to confirm the correct cloning of the inserts into B330 and were termed B330_MUTE_2 and B330_ETO1_807 (Supplementary Table S2). The two final CRISPR constructs are summarized Table 2. A representative T-DNA composition of the CRISPR constructs, targeting either *LpMUTE* or *LpETO1*, is shown in Figure 4E.

### 4.6. DNA Extraction and PCR Confirmation of T-DNA Integration

Genomic DNA was extracted from approximately 5 cm long segments of young and healthy leaves of putative transgenic plants established in the soil using DNA DS Kit (Omega Bio-Tek, Norcross, USA) and the Kingfisher Flex robot (Thermo scientific, Waltham, MA, USA). T-DNA integration was confirmed by PCR amplification of the plant selection marker *hptII* with the primer pairs hptII_F and hptII_R or 252_hpt_fw and 253_hpt_rv yielding amplicons of 799 bp and 780 bp (580 bp for intronless constructs), respectively (Supplementary Table S2). PCRs were performed in a C1000 Touch thermocycler (Biorad, Hercules, CA, USA) in 20 µL consisting of 1× Green GoTaq flexi reaction buffer, 1.5 mM MgCl_2_, 0.2 mM dNTPs, 1.25 U GoTaq G2 polymerase (Promega, Madison, WI, USA), 0.2 µM primers (Microsynth AG, Balgach, Switzerland), and 50 to 75 ng DNA template with the following conditions: 120 s of initial denaturation at 95 °C, 35 cycles of 30 s denaturation at 95 °C, 30 s annealing at 47 °C (hptII_F and hptII_R) or 55 °C (252_hpt_fw and 253_hpt_rv) and 60 s of elongation at 72 °C, 300 s of final elongation at 72 °C. PCR products were run on a 1× TAE 1% (*w*/*v*) agarose gel for about one hour at 70 V before the gels were visualized.

### 4.7. Fluorescence Microscopy

GFP activity was investigated in leaves and shoots of PCR-confirmed transgenic plants. Young and healthy leaves were harvested, and approximately 2 cm long segments 2 to 4 cm below the shoot tips were cut into 0.5 to 2 mm wide leaf strips with a razor blade. Leaf strips were mounted with distilled water onto a microscope slide. Roots were sampled, washed and approximately 1 cm long root tips were mounted with distilled water onto a microscope slide. The prepared samples were then directly observed with an Axio Imager Z1 (Zeiss, Oberkochen, Germany) fluorescence microscope equipped with Zeiss HF (Bright Field) and Zeiss Fs 38 high efficiency (GFP) filters. Exposure of 1500 and 100 ms for the GFP channel and 170 and 80 ms for the Bright Field channel was applied for leaf and root samples, respectively.

### 4.8. GUS Assay

Flowering plants were assessed for GUS activity in leaf, spikelet, stigma, and anther tissue. The GUS activity was determined by incubating tissue samples in GUS assay solution (50 mM NaCl, 1 mM Tris, 1 mg·L^−1^ X-Gluc (Thermo Fisher Scientific, Waltham, MA, USA) and 0.1% (*v*/*v*) Triton X-100) for 12 to 16 h. The samples were then washed and de-stained multiple times with 70% (*v*/*v*) ethanol.

## Figures and Tables

**Table 1:** Outcome of the various transformations performed in this study.

Construct ID	*A. tumefaciens* Strain	Size [bp] ^1^	Genotype	Explant for Callus Induction	Number of Calli/Transformation	Regenerated Plants/Events	PCR Positive (Transgenic) Plants/Events	Percentage of Calli Regenerating Transgenic Events
G150	LBA4044	4564	S23 Z ^2^	shoot tips	60	15/15	15/15	25.0
G151	LBA4044	4564	S23 Z ^2^	shoot tips	60	19/19	19/19	31.7
G152	LBA4044	4564	S23 Z ^2^	shoot tips	60	6/6	6/6	10.0
G153	LBA4044	4564	S23 Z ^2^	shoot tips	72	10/10	10/10	13.9
G154	LBA4044	4564	S23 Z ^2^	shoot tips	60	16/16	16/16	26.7
G155	LBA4044	4564	S23 Z ^2^	shoot tips	72	23/23	23/23	31.9
G156	LBA4044	4564	S23 Z ^2^	shoot tips	60	12/12	12/12	20.0
G157	LBA4044	4564	S23 Z ^2^	shoot tips	72	22/22	22/22	30.6
G158	LBA4044	4564	S23 Z ^2^	shoot tips	60	12/12	12/12	20.0
G159	LBA4044	4564	S23 Z ^2^	shoot tips	72	14/14	14/14	19.4
G160	LBA4044	6349	S23 Z ^2^	shoot tips	36	20/20	20/20	55.6
G161	LBA4044	6335	S23 Z ^2^	shoot tips	36	14/14	14/14	38.9
G162	LBA4044	6362	S23 Z ^2^	shoot tips	36	4/4	4/4	11.1
G163	LBA4044	6308	S23 Z ^2^	shoot tips	36	8/8	8/8	22.2
G164	LBA4044	6280	S23 Z ^2^	shoot tips	36	15/15	15/15	41.7
G166	LBA4044	5593	S23 Z ^2^	shoot tips	36	3/3	3/3	8.3
C801_GFP	GV3101::pMP90RK	5415	S23 Z ^2^	shoot tips	36	11/11	11/11	30.6
C801_GFP	GV3101::pMP90RK	5415	Arolus ^3^	seeds	24	3/2	1/1	4.2
B330_MUTE_2	GV3101::pMP90RK	9446	Arolus ^3^	seeds	192	83/42	70/36	18.8
B330_ETO1_807	GV3101::pMP90RK	9446	6–10 ^4^	anthers	144 ^5^	10/2	9/1 ^5^	0.7
Total					1260	320/270	304/262	20.8

^1^ Size of T-DNA (left and right borders included); ^2^ In vitro maintained clones of S23 Z; ^3^ Selected callus lines with high regeneration potential and low albinism frequency; ^4^ Callus population (mixed origin due to multiple microspores); ^5^ DH transgenic lines.

**Table 2:** Various constructs were used for *Agrobacterium*-mediated transformation in this study. For each construct ID, the *Agrobacterium tumefaciens* strain, the vector backbone, the bacterial selection marker, and the cloning method used are presented. The multigene binary cassette comprised a plant selection cassette expressing hygromycin B resistance (*hptII*) in each construct. The additional gene cassette composition for each construct is given in the “insert” column.

Construct ID	*A. tumefaciens* Strain	Insert	Backbone	Selectable Marker	Cloning Method
G150	LBA4044	p35S::dsRNAbp-hp::t35S	pAGM4673	kanamycin	Golden Gate
G151	LBA4044	plolp2::dsRNAbp-hp::t35S	pAGM4673	kanamycin	Golden Gate
G152	LBA4044	p35S::SDUF247-I-hp::t35S	pAGM4673	kanamycin	Golden Gate
G153	LBA4044	plolp2::SDUF247-I-hp::35S	pAGM4673	kanamycin	Golden Gate
G154	LBA4044	p35S::SDUF247-II-hp::t35S	pAGM4673	kanamycin	Golden Gate
G155	LBA4044	plolp2::SDUF247-II-hp::t35S	pAGM4673	kanamycin	Golden Gate
G156	LBA4044	p35S::ZDUF247-I-hp::t35S	pAGM4673	kanamycin	Golden Gate
G157	LBA4044	plolp2::ZDUF247-I-hp::t35S	pAGM4673	kanamycin	Golden Gate
G158	LBA4044	p35S::ZDUF247-II-hp::t35S	pAGM4673	kanamycin	Golden Gate
G159	LBA4044	plolp2::ZDUF247-II-hp::t35S	pAGM4673	kanamycin	Golden Gate
G160	LBA4044	pdsRNAbp::GUS::tNOS	pCambia1305.1	kanamycin	restriction enzyme
G161	LBA4044	pSDUF247-I::GUS::tNOS	pCambia1305.1	kanamycin	restriction enzyme
G162	LBA4044	pSDUF247-II::GUS::tNOS	pCambia1305.1	kanamycin	restriction enzyme
G163	LBA4044	pZDUF247-I::GUS::tNOS	pCambia1305.1	kanamycin	restriction enzyme
G164	LBA4044	pZDUF247-II::GUS::tNOS	pCambia1305.1	kanamycin	restriction enzyme
G166	LBA4044	p35S::GUS::tNOS	pCambia1305.1	kanamycin	none
C801_GFP	GV3101::pMP90RK	pZmUbi-1::GFP::tOCS	C801p6o2x35s-pZmUbi-1-GFP	spectinomycin	none
B330_MUTE_2	GV3101::pMP90RK	pOsU6::MUTE_2sgRNA	B330p6i2xoR-UcasW-pOsU6	spectinomycin	restriction enzyme
B330_ETO1_807	GV3101::pMP90RK	pOsU6::ETO1_807sgRNA	B330p6i2xoR-UcasW-pOsU6	spectinomycin	restriction enzyme

**Table 3:** Summary of the genes of interest (GOI) used in this study. For each gene, the gene annotation in Italian ryegrass (*Lolium multiflorum* Lam.), the gene name of the orthologous genes in *Oryza sativa* L. subsp. Japonica and a description of the function derived from the gene ortholog in the genus *Oryza* (NCBI, taxid 4527) are presented. If no annotation was present within Italian ryegrass, the position on the scaffold is given.

Gene Name	Gene Annotation i n *Lolium multiflorum* ^1^	Gene Orthologs in *Oryza sativa* subsp. Japonica	Gene Description NCBI
*dsRNAbp*	*Lmu01_818G0000140*^2^ & *Lmu01_1212G0000450*	*Os05g0150400*	Double-stranded RNA-binding protein 2
*SDUF247-I*	*Lmu01_818G0000190*^2^ & *Lmu01_1212G0000400*	*Os05g0198100*	DUF247; Plant protein of unknown function
*SDUF247-II*	*Lmu01_818G0000210* ^2^ & Scf3448 4724980..4726635 ^3^	*Os05g0197900*	DUF247; Plant protein of unknown function
*ZDUF247-I*	*Lmu01_1905G0001490* & *Lmu01_3448G0000640* ^2^	*Os04g0647425*	DUF247; Plant protein of unknown function
*ZDUF247-II*	*Lmu01_1905G0001500*^2^ & Scf34481166016..1167647 ^3^	not annotated	DUF247; Plant protein of unknown function
*MUTE*	*Lmu01_7136G0001450* & *Lmu01_2560G0000140*	*Os03g0294700*	Ethylene-overproduction protein1
*ETO1*	*Lmu01_1530G0000400* & *Lmu01_16G0000470*	*Os05g0597000*	DNA binding protein

^1^ From Copetti et al. [6], ^2^ Gene annotation used for the determination of the promoter region, ^3^ The position within a scaffold of a GOI is given as no annotation is present.

## Data Availability

Not applicable.

## Supplementary Materials

The following supporting information can be downloaded at: www.mdpi.com/article/10.3390/plants11152054/s1, Supplementary Table S1: Summary of the perennial ryegrass (*Lolium perenne* L.) transformation reported; Supplementary File S1: Characterization of editing events; Supplementary File S2: Detailed protocol for callus induction and *Agrobacterium*-mediated transformation; Supplementary Table S2: Primers used in this study.
