# Supplementary material for: Callus Induction from Diverse Explants and Genotypes Enables Robust Transformation of Perennial Ryegrass (Lolium perenne L.)
Source: Plants (Basel). 2022 Aug 5;11(15):2054. doi: 10.3390/plants11152054 (PMC9370183; doi:10.3390/plants11152054)
Supplement: Supplementary file 1 [file plants-11-02054-s001.zip › Supplementary_File_S1.pdf]

Supplementary File S1: Characterization of editing events

Detection of edits by Sanger sequencing

A 489 bp long *LpMUTE* amplicon containing the MUTE_2 sgRNA binding site was amplified in 50 µL containing 1X Q5 reaction buffer, 0.2 mM dNTPs, 0.2 µM of primers MUTE_F1 and MUTE_R3 (**Supplementary Table S2**), 1 U Q5® high-fidelity DNA polymerase (New England Biolabs, Ipswich, USA) and 75 ng of DNA template. A 1171 bp long *LpETO1* amplicon containing the ETO1_807 sgRNA binding site was amplified in 50 µL containing 1X Q5 reaction buffer, 0.2 mM dNTPs, 0.2 µM of primers ETO1_F and ETO1_R (**Supplementary Table S2**), 1 U Q5® high-fidelity DNA polymerase (New England Biolabs, Ipswich, USA) and 75 ng of DNA template. For *LpMUTE*, cycling conditions were 30 sec at 98 °C, followed by 35 cycles of 10 sec at 98 °C, 30 sec at 66 °C and 30 sec at 72°C, and a final elongation step at 72 °C for 2 min. For *LpETO1*, cycling conditions were 30 sec at 98 °C, followed by 35 cycles of 10 sec at 98 °C, 30 sec at 63 °C and 40 sec at 72 °C, and a final elongation step at 72 °C for 2 min. All PCR products were separated on an agarose gel (1 % agarose 1X TAE at 80 V for 1 h) to confirm the correct size of the amplicons before they were purified using QIAquick Gel Extraction Kit (Qiagen, Hilden, Germany). The purified PCR products were sent for Sanger sequencing (Microsynth AG, Balgach, Switzerland) with primer MUTE_F2 for *LpMUTE* and primer ETO1_F for *LpETO1* (**Supplementary Table S2**). In case of chromatograms with superimposed signals in proximity to the sgRNA binding sites, TIDE (Tracking of Indels by DEcomposition), was used to characterize the induced mutations.

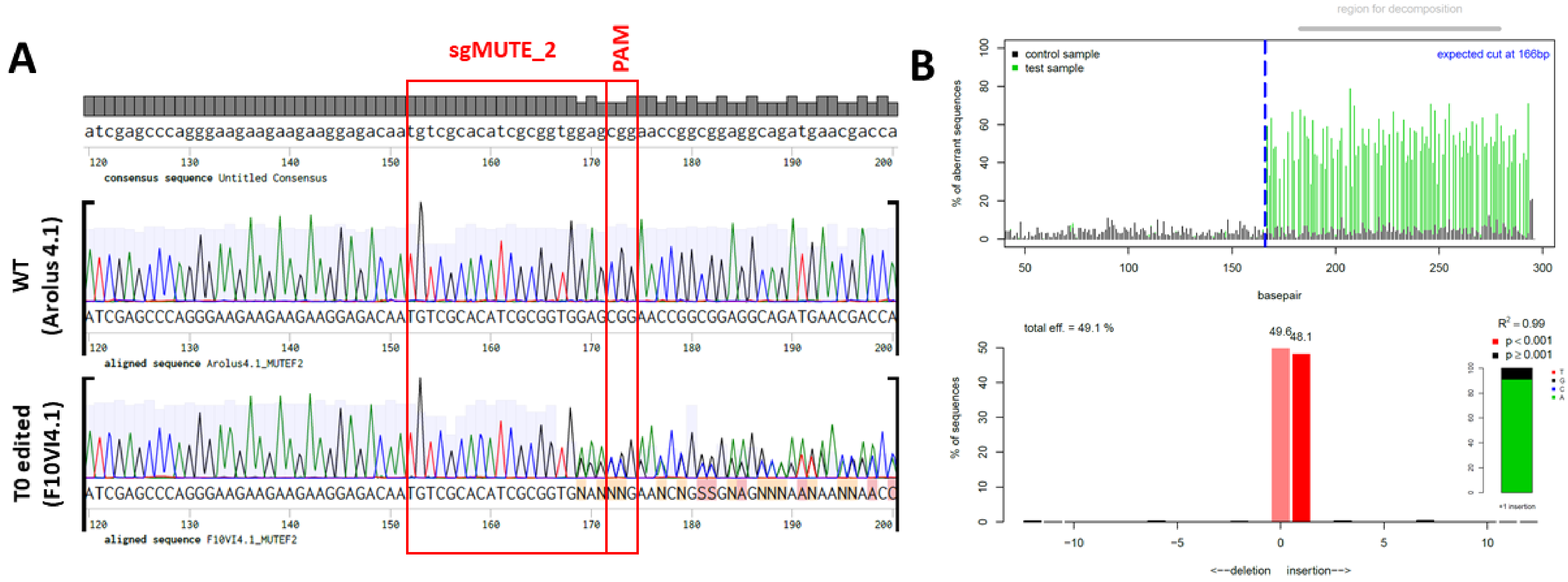

Figure SF1. Detection of insertions or deletions (indels) in B330_MUTE_2 transgenic lines. (A) Chromatograms showing the *LpMUTE* region containing the sgMUTE_2 binding site (WT and T0 edited lines). In the T0 edited chromatogram, superimposed signals typically resulting from indels can be observed starting 3 bp upstream of the protospacer adjacent motif (PAM); (B) TIDE analysis of the edited events identified. Frequencies of edited and WT alleles of approximately 50 % indicate the heterozygous nature of the edit. + 1 bp insertion is predicted to be an Adenine. Test sample was F10VI4.1 and control sample Arolus 4.1

RT-qPCR assays

RNA extraction and cDNA synthesis

Approximately 4 cm long young and healthy leaves segments from four different tillers of the same plant were harvested between 8 am and 10 am. The harvested material was placed in 2 mL screw cap tubes (OMNI International inc., Kennesaw, USA) with 2 mm diameter metallic beads and was snap frozen in liquid nitrogen. The harvested samples were stored at - 80 °C for a maximum of two weeks before they were further processed. Frozen samples were ground for 20 s at 3.1 $m \cdot s^{-1}$ using an Omni Bead Ruptor 24 (OMNI International inc., Kennesaw, USA) and total RNA was extracted using TRIzol™ Reagent (Thermo Fisher Scientific, Waltham, USA) following the manufacturer's instructions. Total RNA recovered from the extraction was DNAse treated using the DNA-*free*™ DNA Removal Kit (Thermo Fisher Scientific, Waltham, USA) according to the supplier's instructions. Total RNA was quantified using a Qubit Fluorometer and the RNA Broad Range Assay Kit (Thermo Fischer Scientific, Waltham, USA) and its quality assessed using an Agilent 2100 Bioanalyzer (Agilent, Santa Clara, USA). Finally, 5 µg of total RNA were converted into cDNA with random hexamers using the Maxima H Minus Reverse Transcriptase (Thermo Fisher Scientific, Waltham, USA) according to the manufacturer's protocol.

Cas9 expression

The relative *Cas9* expression was determined according to Pfaffl [58] using *EF1α* [59] as reference gene and F8AI1 as control sample. Controls consisting of 5 ng of total RNA (no RT) were used to confirm the absence of genomic DNA contamination. All 96-well reactions plates were prepared using the automated liquid handling PIPETMAX® device and for each sample reactions were performed in duplicates. RT-qPCRs were performed in 0.1 mL MicroAmp Fast 96-Well Reaction plates (Applied Biosystems, California, USA) with a 7500 Fast Real-Time PCR System (Applied Biosystems, California, USA) device using the 7500 software v2.0.6. All reactions were performed in 20 µL with 1X HOT FIREPol® EvaGreen® qPCR Mix Plus (Solis BioDyne, Tartu, Estonia) with 0.1 µM of Cas9WT_2_F and Cas9WT_2_R primers (**Supplementary Table S2**) and 1 µL cDNA as template. Cycling conditions consisted of an initial denaturation step of 12 min at 95 °C followed by 40 cycles consisting of 15 s at 95 °C, 20 s at 58 °C and 1 min at 72 °C.

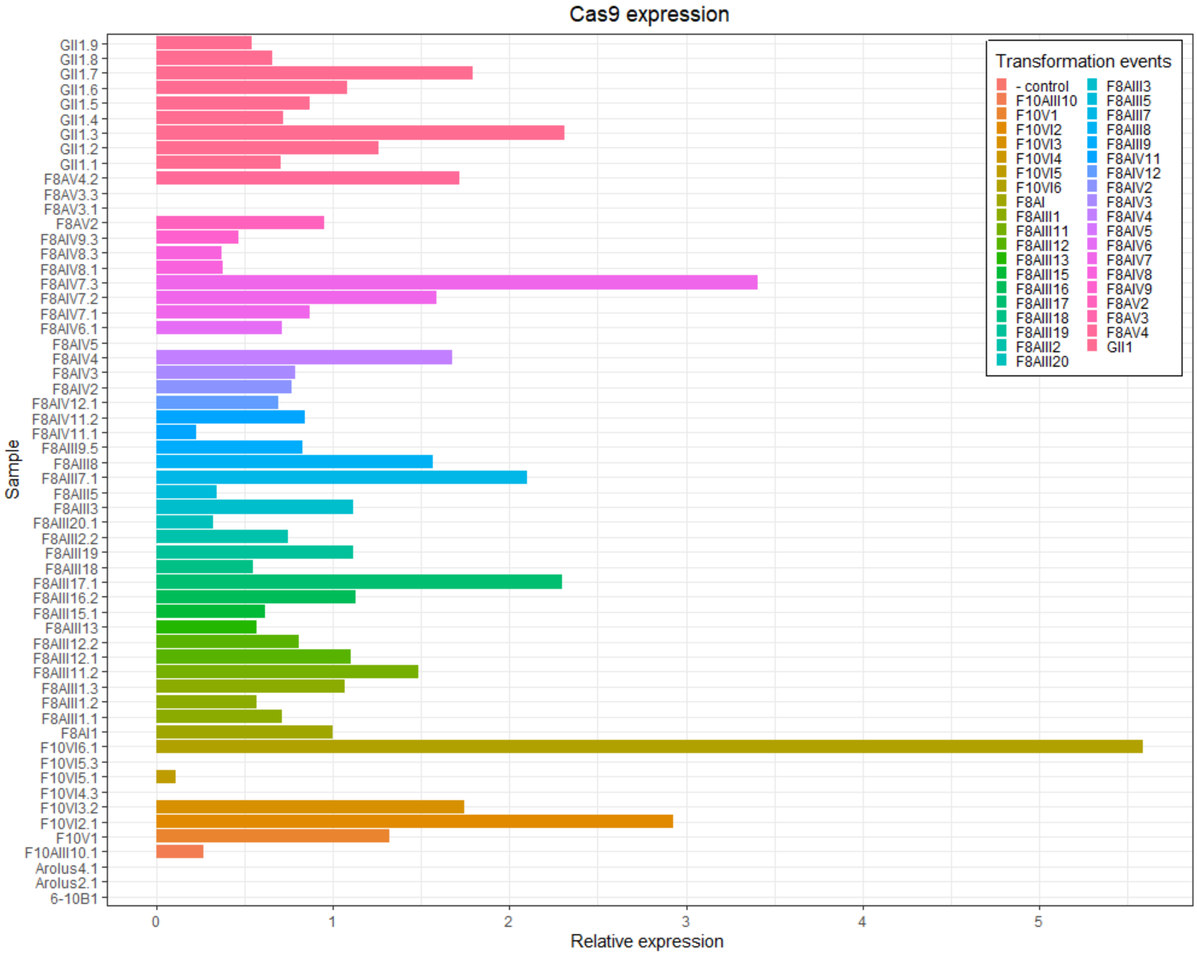

Figure SF2. Mean *Cas9* expression values in a subset of transgenic plants measured in preliminary RT-qPCR experiments. Expression is relative to the *Cas9* expression from the reference sample F8AI1. Colors indicate transformation events. Arolus 2.1, Arolus 4.1 and 6-10 B1 are untransformed controls.
