# Supplementary material for: Callus Induction from Diverse Explants and Genotypes Enables Robust Transformation of Perennial Ryegrass (Lolium perenne L.)
Source: Plants (Basel). 2022 Aug 5;11(15):2054. doi: 10.3390/plants11152054 (PMC9370183; doi:10.3390/plants11152054)
Supplement: Supplementary file 1 [file plants-11-02054-s001.zip › Supplementary_File_S2.pdf]

Supplementary File S2: Detailed protocol for callus induction and *Agrobacterium*-mediated transformation

Media composition and preparation

The composition and usage of the media used in this study are described in **Table SF1**. All chemicals were ordered at Duchefa (Duchefa Biochemie B.V., Haarlem, Netherlands) except hygromycin B (Carl Roth, Karlsruhe, Germany), acetosyringone (Sigma-Aldrich, St-Louis, USA), ferrous sulfate chelate solution 100X (Sigma-Aldrich, St-Louis, USA), Pluronic F68 (Thermo Fisher Scientific, Waltham, USA), Timentin (Melford, Suffolk, U.K.) and Meropenem (Melford, Suffolk, U.K.). Modified 190-2 salts were prepared according to Begheyn et al. [60].

Media were prepared by dissolving chemicals in double distilled (ddH$_2$O) or Milli-Q® water (Merck, Darmstadt, Germany), in 80 % of the final volume, pH was adjusted with 1 M NaOH or HCl and final volume was set. Media were sterilized by autoclaving for 15 min at 121 °C and solidified media were obtained by adding 3.5 g·L^{-1} Gelrite prior to autoclaving. Antibiotics, heat-sensitive plant growth regulators (e.g., BAP) or other additives (e.g., acetosyringone and Pluronic F68) were added from filter-sterilized stocks to the desired concentration after autoclaving, when media had cooled down to approximately 60 °C.

Media supplemented with antibiotics were named according to their original name and the concentration of the added antibiotics (e.g., MSO and MSO + H50 + C100 when supplemented with 50 mg·L^{-1} hygromycin and 100 mg·L^{-1} carbenicillin). As different selection regimes were applied, the detailed protocol below does not inform about the antibiotic supplemented to selection media but **Table SF1** indicates different antibiotics concentrations used.

Table SF1. Composition and usage of the media used in this study.

Name	Uses	Composition	Antibiotics	pH
MSO	In vitro plant maintenance and rooting	MS including modified vitamins (Duchefa M0245), 3 % (w/v) sucrose	25/80 mg·L^{-1} hygromycin B, 250 mg·L^{-1} carbenicillin, 200 mg·L^{-1} Timentin	5.6
R2M	Callus induction (anthers)	Modified 190-2 salts, McCown woody plant vitamins (Duchefa M0408), 0.5 X ferrous sulfate chelate, 9 % (w/v) maltose, 1.5 mg·L^{-1} 2,4-D, 0.5 mg·L^{-1} kinetin	-	6
135MODM	Callus induction (seeds and shoot tips) and propagation	MS (Duchefa M0221), CHU vitamins (Duchefa C0401), 3 % (w/v) maltose, 5 mg·L^{-1} 2,4-D, 0.05 mg·L^{-1} BAP	-	5.6
135MODS	Callus selection	MS including modified vitamins (Duchefa M0245), 3 % (w/v) sucrose, 5 mg·L^{-1} 2,4-D, 0.05 mg·L^{-1} BAP	25/50/70/75 mg·L^{-1} hygromycin B, 250 mg·L^{-1} carbenicillin, 200 mg·L^{-1} Timentin, 50 mg·L^{-1} Meropenem	5.6
RMODM	Regeneration	MS including modified vitamins (Duchefa M0245), 3 % (w/v) maltose, 1 mg·L^{-1} NAA, 1 mg·L^{-1} BAP	50/70 mg·L^{-1} hygromycin B, 250 mg·L^{-1} carbenicillin, 200 mg·L^{-1} Timentin, 50 mg·L^{-1} Meropenem	5.6
Infection medium	*Agrobacterium* infection	MS (Duchefa M0221), CHU vitamins (Duchefa C0401), 6.84 % (w/v) sucrose, 3.6 % (w/v) glucose, 5 mg·L^{-1} 2,4-D, 200 µM acetosyringone, 0.02 % (v/v) Pluronic F68	-	5.2
Co-cultivation medium	*Agrobacterium* co-cultivation	MS (Duchefa M0221), CHU vitamins (Duchefa C0401), 6 % (w/v) maltose, 300 mg·L^{-1} cysteine, 5 mg·L^{-1} 2,4-D, 200 µM acetosyringone	-	5.2

Callus induction from shoot tips

1. Excise the shoot tip meristems from in vitro maintained plantlets using fine forceps, scalpel and binocular microscope under sterile conditions.
2. Plate the explants onto 135MODM (**Table SF1**) and incubate at 24 °C in the dark.
3. After 4 to 6 weeks of culture, calli will be visible. For propagation, subculture further at reduced time intervals onto 135MODM to promote fast growth and grow at 24 °C in the dark. Subculture the calli onto 135MODM for an additional time one week before the calli will be used for *Agrobacterium*-mediated transformation.

Callus induction from seeds

1. Place 0.2 to 0.5 g of seeds in 15 mL ddH_2O in a 50 mL Falcon tube and let soak overnight (ON) at 4 °C in the dark.
2. The next day, dehusk the seeds by ''peeling'' them using forceps and binocular microscope under non-sterile conditions.
3. Clean the dehusked seeds with 50 mL 94 % (v/v) EtOH supplemented with a few drops of Tween 20 (Sigma-Aldrich, St-Louis, USA) in a 50 mL Falcon tube for 1-2 min. Then surface-sterilize the dehusked seeds with 50 mL ± 5 % (v/v) NaOCl supplemented with a few drops of Tween 20 in a 50 mL Falcon tube for around 45 min in a horizontal shaker at room temperature. From now on, work under sterile conditions. Wash seeds three times with 50 mL autoclaved ddH2O. Plate the seeds onto three 85 mm diameter dry and sterile filter papers placed in a 100 mm diameter petri dish and leave them open to dry in front of the airflow for 30 to 45 min.
4. Plate the seeds onto 135MODM and incubate in the dark at 24 °C for 4 to 6 weeks.
5. Inspect the plates twice a week to check for potential bacterial and fungal contaminations and to excise germinating shoots.
6. After 4 to 6 weeks of culture calli will be visible. Maintain calli induced from single seed separately to ensure working with single genotypes, also referred to as callus lines. For propagation, subculture further at reduced time intervals onto 135MODM to promote fast growth and grow at 24 °C in the dark. Select callus lines displaying rapid growth and high spontaneous regeneration on 135MODM for transformation. Subculture the calli onto 135MODM for an additional week before the calli will be used for *Agrobacterium*-mediated transformation.

Agrobacterium-mediated transformation of calli of perennial ryegrass

Preparation of *Agrobacterium tumefaciens*

1. Use glycerol stocks previously prepared from single colonies of *A. tumefaciens* harboring the binary vectors of interest to inoculate 200 to 300 mL of lysogeny broth supplemented with adequate antibiotics in sterile 500 mL Erlenmeyer flask. Grow at 28 °C and 200 rpm in an incubator-shaker until optical density at 600 nm (OD_{600}) has reached 0.7 to 1.
2. Transfer *A. tumefaciens* cultures to 50 mL Falcon tubes and centrifuge at 3000 to 3500 rpm for 10 min at room temperature. Carefully remove the supernatant and resuspend the bacterial pellet in infection medium (**Table SF1**) to an OD_{600} of 0.7 to 1. Grow for 4 h at 28 °C and 200 rpm in an incubator-shaker.

Agrobacterium-mediated transformation: infection, co-cultivation, wash and selection

3. Transfer calli from petri dishes to 50 mL Falcon tubes (one plate per Falcon tube, should represent around 3 to 4 mL of calli) and add 2 to 3 mL of infection medium (**Table SF1**) to wet the calli.
4. Immerse the 50 mL Falcon tubes in a water bath at 43 °C for 3 min. Add 10 to 25 mL of *Agrobacterium* cultures previously prepared per tube. Mix gently by inverting and leave to stand for 30 min at R.T.
5. Pour off the *Agrobacterium* cultures and plate the calli onto three 85 mm diameter dry and sterile filter papers placed in a 100 mm diameter petri dish. Leave the petri dish open to dry in front of the airflow in the sterile bench for 30 to 60 min.
6. Transfer calli onto three 85 mm diameter sterile filter papers wetted with 3 mL co-cultivation medium (**Table SF1**) placed in a new 100 mm diameter petri dish. Seal the petri dishes with parafilm and incubate at 24 °C in the dark for 3 to 4 days.
7. After 3 to 4 days of co-cultivation, wash the calli in a 50 mL Falcon tube with 30 to 40 mL ddH_2O + C500. Mix gently by inverting the tubes a few times and pour off the liquid. Plate the calli onto three 85 mm diameter dry and sterile filter papers placed in a 100 mm diameter petri dish. Leave the petri dish open to dry in front of the airflow in the sterile bench for 30 to 60 min. This washing step is optional but is helpful against *Agrobacterium* overgrowth in the regeneration and rooting steps.
8. Plate the calli onto 135MODS + antibiotics (**Table SF1**) and incubate at 24 °C in the dark for 4 to 6 weeks.

Plant regeneration, rooting and transfer to the soil

9. After 4 to 6 weeks of selection, transfer onto RMODM + antibiotics (**Table SF1**) to promote shoot regeneration and incubate at 24 °C under long day conditions (16 h light: 8 h dark).
10. Transfer developing shoots (≥ 2 cm long) withstanding the selection onto MSO + antibiotics (**Table SF1**) for rooting and further incubate at 24 °C under long day conditions (16 h light: 8 h dark).
11. After 4 to 6 weeks of rooting onto MSO + antibiotics, transfer the single putative transgenic plantlets that developed roots into the soil. Grow at ±20 °C under long day conditions (16 h light: 8 h dark) for rapid growth.
