# Supplementary material for: Callus Induction from Diverse Explants and Genotypes Enables Robust Transformation of Perennial Ryegrass (Lolium perenne L.)
Source: Plants (Basel). 2022 Aug 5;11(15):2054. doi: 10.3390/plants11152054 (PMC9370183; doi:10.3390/plants11152054)
Supplement: Supplementary file 1 [file plants-11-02054-s001.zip › Supplementary_Table_S1.pdf]

Supplementary Table S1. Summary of the perennial ryegrass (*Lolium perenne* L.) transformation reported. Seeds (S), shoot tips (ST), immature embryo (IE), inflorescence (II), axillary bud (AB), meristematic region (MR) and meristem (M).

Reference	Explant	Genotype	Transformation method	*A. tumefaciens* strain
van der Maas et al. (1994) [13]	S	Aurora	biolistic	-
Spangenberg et al. (1995) [14]	S	Citadel	biolistic	-
Wang et al. (1997) [15]	NA	Lp 8-1, Lp 6-1	direct gene transfer	-
Dalton et al. (1998) [16]	ST	S23 Z	silicon carbide fiber	-
Dalton et al. (1999) [17]	ST	S23 Z	biolistic	-
Altpeter et al. (2000) [18]	S, IE, II	Limes, Aurora, L 6, Lisabelle	biolistic	-
Xu et al. (2001) [19]	S	NA	biolistic	-
Altpeter et al. (2004) [20]	NA	NA	biolistic, *A. tumefaciens*	AGL1
Hisano et al. (2004) [21]	S	Riikka	biolistic	-
Petrovska et al. (2004) [22]	S	Kelvin	biolistic	-
Chen et al. (2005) [23]	S	Majesty, Lisabelle	biolistic	-
Wu et al. (2005) [24]	S	Topgun	*A. tumefaciens*	LBA4404, EHA105
Altpeter et al. (2006) [25]	AB	Limes	*A. tumefaciens*	AGL0
Bajaj et al. (2006) [26]	MR	Tolosa	*A. tumefaciens*	EHA101
Cao et al. (2006) [27]	S	Medalist Gold	*A. tumefaciens*	EHA105
Petersen et al. (2006) [28]	M	F6	biolistic	-
Sato et al. (2006) [29]	ST	Norlea	*A. tumefaciens*	EHA101
Wu et al. (2007) [30]	S	Topgun	*A. tumefaciens*	NA
Gadegaard et al. (2008) [31]	M	N2, F6	biolistic	-
Li et al. (2011) [32]	NA	NA	*A. tumefaciens*	NA
Wu et al. (2012) [33]	S	NA	*A. tumefaciens*	AGL1
Patel et al. (2013) [34]	S	Citation IV	*A. tumefaciens*	EHA105
Zhang et al. (2013) [35]	S	MontereyII	*A. tumefaciens*	EHA105
Vail et al. (2015) [36]	NA	NA	*A. tumefaciens*	AGL1
Cen et al. (2016) [37]	S	Citation IV	*A. tumefaciens*	EHA105
Panter et al. (2017) [38]	S	FLp418-20	biolistic	-
Badenhorst et al. (2018) [39]	S	FLp418-20	biolistic	-
Xu et al. (2019) [40]	NA	NA	*A. tumefaciens*	AGL1
Esmaeili et al. (2019) [41]	S, ST	Grassland, Numan	*A. tumefaciens*	LBA4404, GV3101
Dalton (2020) [42]	ST	S23 Z	*A. tumefaciens*	AGL1
Zhang et al. (2020) [43]	S	Goyave	biolistic	-
Cooney et al. (2021) [44]	II	Alto, Grassland Impact	biolistic, *A. tumefaciens*	NA
Hang et al. (2021) [45]	S	Citation Fore	*A. tumefaciens*	LBA4404
Yu et al. (2021) [46]	NA	NA	*A. tumefaciens*	NA
Beechey-Gradwell et al. (2022) [47]	II	Alto, Grassland Impact	biolistic, *A. tumefaciens*	NA
